# Role of Innate Immune Response in Non-Alcoholic Fatty Liver Disease: Metabolic Complications and Therapeutic Tools

**DOI:** 10.3389/fimmu.2014.00177

**Published:** 2014-04-23

**Authors:** Rosaria Meli, Giuseppina Mattace Raso, Antonio Calignano

**Affiliations:** ^1^Department of Pharmacy, University of Naples “Federico II”, Naples, Italy

**Keywords:** NAFLD, insulin resistance, pathogen recognition receptors, innate immune cells, cytokines, inflammation, DAMPs, pathogen-associated molecular patterns

## Abstract

Non-alcoholic fatty liver disease (NAFLD) is currently the most common liver disease worldwide, both in adults and children. It is characterized by an aberrant lipid storage in hepatocytes, named hepatic steatosis. Simple steatosis remains a benign process in most affected patients, while some of them develop superimposed necroinflammatory activity with a non-specific inflammatory infiltrate and a progression to non-alcoholic steatohepatitis with or without fibrosis. Deep similarity and interconnections between innate immune cells and those of liver parenchyma have been highlighted and showed to play a key role in the development of chronic liver disease. The liver can be considered as an “immune organ” because it hosts non-lymphoid cells, such as macrophage Kupffer cells, stellate and dendritic cells, and lymphoid cells. Many of these cells are components of the classic innate immune system, enabling the liver to play a major role in response to pathogens. Although the liver provides a “tolerogenic” environment, aberrant activation of innate immune signaling may trigger “harmful” inflammation that contributes to tissue injury, fibrosis, and carcinogenesis. Pathogen recognition receptors, such as toll-like receptors and nucleotide oligomerization domain-like receptors, are responsible for the recognition of immunogenic signals, and represent the major conduit for sensing hepatic and non-hepatic noxious stimuli. A pivotal role in liver inflammation is also played by cytokines, which can initiate or have a part in immune response, triggering hepatic intracellular signaling pathways. The sum of inflammatory signals and deranged substrate handling induce most of the metabolic alteration traits: insulin resistance, obesity, diabetes, hyperlipidemia, and their compounded combined effects. In this review, we discuss the relevant role of innate immune cell activation in relation to NAFLD, the metabolic complications associated to this pathology, and the possible pharmacological tools.

## Introduction

Non-alcoholic fatty liver disease (NAFLD) represents a wide range of pathologies beginning with simple triglyceride accumulation inside the hepatocytes, a benign condition that can evolve and progress to non-alcoholic steatohepatitis (NASH), characterized by inflammation and hepatocytes injury. Disease progression occurs together with metabolic and inflammatory derangements that accompanied by genetic and environmental factors, promote a persistent activation of the immune system. A complex backdrop involves adipokines, metabolites [i.e., free fatty acids (FFAs)], cell-derived fragments, all released by damaged cells and metabolic disrupted organs, such as liver and adipose tissue (1, 2). The innate immune system that in physiological condition maintains tissue and organ homeostasis, may undergo an aberrant activation, and trigger harmful inflammation, which contributes to initially low-grade inflammation, tissue and organ injury, and lately fibrosis and carcinogenesis (3). A key role in the pathogenesis of NAFLD is played by gut-derived endotoxin, a component of the Gram-negative bacteria wall, which reaches the liver when the colonic mucosa is disrupted. Together with lipopolysaccharide (LPS), other bacterial products, such as lipoproteins, flagellins, and peptidoglycans termed pathogen-associated molecular patterns (PAMPs), share or not similar molecular structures.

Pathogen recognition receptors (PRRs), such as toll-like receptors (TLRs) and nucleotide oligomerization domain (NOD)-like receptors (NLRs), are responsible for the recognition of immunogenic signals, representing a major conduit for alterations in liver. The various types of TLRs enables to discriminate between different PAMPs that belong to several microbial classes For instance, TLR4 is able to recognize LPS from Gram-negative bacteria, while TLR2 detects Gram-positive bacteria through the recognition of cell membrane components, i.e., lipoteichoic acid, peptidoglycan, and various lipopetides and lipoproteins, while other TLRs, such as 3 and 7, sense viral infection binding double- and single-stranded RNA (4). Therefore, these receptors recognized more than one ligand, binding even completely structural different molecules (5). The activation of TLRs does not occur in physiological conditions, conversely, when an environmental change induces tissue injury and cell dying, endogenous protein, and non-protein ligands, named damage-associated molecular pattern (DAMPs) or alarmins (6) can bind to TLRs and induce their activation. DAMP sources include heat shock proteins, high mobility group box 1, breakdown products of the extracellular matrix (i.e., hyaluronan, fibrinogen, and fibronectin), and non-protein substrates (5–7). Also NLRs are able to recognize PAMPs and DAMPs, and are expressed mainly on antigen-presenting cells and epithelial cells. NLRs activation leads to the assembly of the inflammasome, containing caspase-1, whose activation leads to inflammation and apoptosis. Caspase-1, in fact is also known as interleukin cleavage enzyme, responsible for the conversion of prointerleukin-1, -18, and -33 in the respective mature forms. The activation of PRRs leads to cytokine production, contributing to liver injury and metabolic complications. In particular TNFα and IL-6, originally considered classical inflammatory cytokines, are now considered major links between steatosis, insulin resistance (IR), and related inflammatory disorders. Indeed, it has been demonstrated that TNFα reduced insulin signaling activation (8) and its expression in liver is enhanced in patients affected by NAFLD (9, 10). Also IL-6 has been implicated in IR, in fact is now considered its predictor or pathogenetic marker. Moreover its expression correlates with the degree of hepatic inflammation, and fibrosis (9, 11). Recent studies in mouse models of NASH implicate caspase-1 and inflammasome in inflammatory response associated to metabolic complications (12, 13). Therefore, metabolic systems are closely integrated with downstream pathway of TLRs and NLRs. Upon pathogens sensing by the innate immune system, concomitantly insulin signaling and inflammatory response are modulated as a result of the activation of PPRs pathways, triggering both immunological and metabolic processes (14, 15).

## Gut–Liver Axis

The anatomical site and cellular architecture of the liver make it a key organ in metabolic essential functions. The position between gut and systemic circulation, guarantees that all the substances orally ingested and absorbed by the intestine have to necessarily pass to the liver. As a result, the liver has developed the ability not only to receive, process, and store substances, as it occurs in case of nutrients, but also to respond to exogenous antigenic molecules (i.e., food, viral, bacterial, and parasitic substances). Therefore, the liver, originally considered only a metabolic organ, is now recognized as a mediator of systemic and local innate and adaptive immunity. Indeed, it is continuously exposed to antigens from the gut through the portal vein since sinusoidal endothelial cells of liver parenchyma are characterized by the lack of the basement membrane. Moreover, the liver contains a specialized cellular repertoire able to degrade and remove toxins, exogenous antigens, and infectious agents. This expertise allows to achieve an immune tolerance, avoiding overactivation of immune system or, conversely, to switch the tolerant response to a responsive state when demanded (16–21). Therefore, the status of gut integrity strictly accounts for the exposure of liver to gut microbiota and antigenic food components. Translocation of large amounts of gut-derived products is usually prevented by intact barrier systems provided by intestinal epithelial cells (22). When the disruption of intestinal barrier results in leaky gut, bacteria and bacterial products easily migrate to the mesenteric lymph nodes and to the liver. This bacterial translocation may impair liver homeostasis and trigger liver inflammation, inducing the innate immune response (3, 22, 23).

## Innate Immunity and Cell Involvement in NAFLD: Role of Cytokines

Apart from parenchymal hepatocytes, the liver also contains complex repertoires of lymphoid and non-lymphoid cells, key effectors for hepatic immunoregulation, and defense (16, 21).

Hepatic lymphoid cells comprise resident lymphocytes, distinct both in function and phenotype, from their counterparts in the peripheral circulation and in other organs. In particular, liver hosts conventional (i.e., B cells, CD4+ and CD8+ T cells, natural killer cells) and non-conventional lymphoid cells (i.e., γδ TCR+ T cells, natural killer T cells, CD4− and CD8− T cells). A key role in specific immune function is also exerted by mucosal-associated invariant T cells that are a highly specialized T cell population in the vascular network of the liver (24). Regulatory T cell populations seem to have an important role in maintaining a beneficial balance in the liver between immuno-tolerance and activation (25). In addition to classic parenchymal hepatocytes and cholangiocytes, the liver contains other cell types responsible for the homeostasis of the innate and adaptive immune system. Among the non-lymphoid cells, Kupffer cells (KC) (26) and dendritic cells (27, 28) from the myeloid lineage have a major role in the immune response. Dendritic cells are the primary antigen-presenting cells of the liver. However, cholangiocytes can also act as antigen-presenting cells (29), thus playing an additional role in the hepatic immune function.

## Kupffer Cells

Resident- or monocyte-derived KC are the largest population of mononuclear phagocytes in the body. They are present throughout the liver, but there is variation in the population density, cytological characteristics, and physiologic functions of KC in different zones of the hepatic acinus/lobule (30). During liver injury and diseases, monocytes rapidly differentiate into mature cells that are indistinguishable from genuine KC, independently from the circulating monocytes (31). They are strategically located in liver sinusoids, therefore they come firstly in contact with exogenous immunoreactive material or endogenous signals (32) phagocytosing, processing and presenting antigen, and secreting various pro-inflammatory mediators such as cytokines, prostanoids, nitric oxide, and reactive oxygen intermediates (33). Expression of the Fc receptor results in non-specific phagocytosis of immune complexes as well as antibody-coated particles such as microorganisms and allows KC to have a significant role in control of inflammatory and immunologic processes (34). Moreover, KC also express complement receptors for binding and phagocytosis. Bacterial endotoxin derived from the gut or endotoxin injected in rodents are cleared principally by the liver and taken up primarily by KC (35). Within the liver, LPS binds to LPS-binding protein, which then facilitates the transfer of LPS to CD14 on the surface of KC. Signaling of LPS through CD14 is mediated by the downstream TLR4, resulting in activation of KC and direct involvement of the innate immune system (36). Also TLR2 has a key role in KC response to insults, in fact TLR2-deficient KC showed an impaired response toward microbes, such as *Listeria monocytogenes* (37). After activation, KC can either avoid escalation of inflammatory response, through a fine control of adaptive immune, or vice versa failure to halt inflammation, properly recognizing and eliminating danger molecules.

Generally, KC are exposed to low gut-derived LPS levels. This stimulus allows KC to trigger an escape mechanism that involves IL-10, which in turn contributed to the down-regulation of pro-inflammatory cytokines (38). On the other hand, following massive TLR4 stimulation, KC produce several chemokines and cytokines, such as TNFα, IL-1β, IL-6, IL-12, and IL-18 (39, 40). IL-12 and IL-18, in particular, can induce IFN-γ production by NK cells, facilitating microbial eradication, and hepatic wound healing (41). Thus, KC may have a higher tolerance to LPS, or a prompt inflammatory response, adapting to the contingent circumstances. To maintain the steady state, KC are able to mount opposite responses to exogenous triggers, polarizing to M1 or M2 subphenotypes (42). In response to TLR ligands and cytokines, KCs undergo polarized inflammatory programs, known as M1 (classical) or M2 (alternative) activation. While the M1 phenotype is characterized by the increase in the production of inflammatory cytokines, and reactive nitrogen and oxygen species, associated by microbicidal and tumoricidal activity, M2 macrophages show immunomodulatory functions, parasite containment, and tissue remodeling. Similarly to M1 phenotype, KC can contribute to the pathogenesis of liver disease, increasing the production of pro-inflammatory cytokines (e.g., TNFα) (17, 42, 43). Conversely, adiponectin was recently shown to shift KC polarization to the M2/anti-inflammatory phenotype (44, 45), preventing progression of NASH in mice (46). Adiponectin decrease, as well as adiponectin gene deletion, induces hepatic steatosis progression, fibrosis, and tumor development (47). Moreover, KCs have also metabolic function, regulating fatty acids oxidation, increasing hepatic lipid storage and IR, as mechanisms of adaptation to increased caloric intake (48). This event is triggered by the secretion of inflammatory cytokines (i.e., TNF, IL-6, IL-1β) (48), thus suggesting a beneficial role for alternatively M2-activated KCs in metabolic derangements (49, 50).

## Hepatocytes

Hepatocytes exert metabolic and detoxifying functions, and prompt the acute phase response. They express TLR4, even if high doses of LPS are needed to induce significant effects (51, 52). Their expression of TLR2, rather than TLR4, is up-regulated in inflammatory conditions, suggesting a major responsiveness to TLR2 activation following an insult (52). Hepatocytes are able to clear LPS from systemic circulation, through its uptake and release into the bile, more than KCs, since KC-depleted rats have the same capacity of LPS removal (53). TLR4, CD14, and myeloid differentiation (MD)-2 have an obligatory role for LPS uptake by hepatocytes. Nevertheless, TLR4 signaling does not seem to be required for this process *in vivo* (54).

## Hepatic Stellate Cells

Hepatic stellate cells (HSCs) include around 30% of the non-parenchymal cells (55). In physiological conditions, HSCs are quiescent cells, and represent the largest content of vitamin A in the body (56). When the liver is injured, damaged hepatocytes and immune cells start to release signal molecules that, targeting HSCs, induce their trans-differentiation into activated myofibroblast-like cells (55, 57, 58). Therefore, activated HSCs switch from resting vitamin A-rich cell to proliferating, fibrogenic, and contractile cell (58, 59), leading to hepatic fibrosis. The main activators of their trans-differentiation are platelet-derived growth factor and transforming growth factor (TGF)-β1, produced by activated KC, infiltrating monocytes, platelets, and damaged hepatocytes (60). The resting HSCs may acquire adipogenic or myogenic phenotype during the trans-differentiation (61), determined by adipogenic and myogenic gene expression. In fact, adipogenic genes are down-regulated under ischemia and inflammation and up-regulated by peroxisome proliferator-activated receptor (PPAR)γ. Conversely, activated HSCs, expressing myogenic genes, can develop a myofibroblast-like feature, and release extracellular matrix components, including fibrillar collagens (collagen I and III) (62). Moreover, HSCs can also produce tissue inhibitors of metalloproteinases, which may reduce extracellular matrix components degradation, decreasing matrix metalloproteinases (MMPs) activities.

In addition to TGF-β1, other factors are implicated in HSCs activation to become myofibroblastic, such as the Hedgehog (Hh) pathway (63), cytokine stimulation (particularly TNF-α, IL-1β, and IL-6) (64), and leptin (65). Leptin, a well-recognized pro-fibrotic hormone, activates JAK2/STAT3 pathway following the binding of its hepatic functional receptor, and increases the expression of tissue inhibitors of metalloproteinases, leading to matrix deposition. Moreover, it also inhibits matrix degradation, reducing MMPs expression (66). In fact, leptin receptor expression is low in quiescent HSCs, but increases during their transdifferentiation (67). Conversely, adiponectin released from adipose tissue reduces HSC migration and proliferation (67). Accumulating evidence indicates that a role in HSC activation is also played by renin–angiotensin system expressed in injured liver. In particular, HSCs generate *de novo* angiotensin 2 (68) that increases HSC proliferation and migration, cytokine and collagen synthesis (69, 70). Finally, TLR4 contributes to the activation of HSCs through an MyD88–NF-κB-dependent pathway (3, 71). HSCs activation in NASH have been already reported (72), however, more studies are needed to better clarify its role in NAFLD onset and progression.

## Biliary Epithelial Cells

Biliary epithelial cells lined the biliary tree, which carries the bile into the intestine. As for HSCs, recently it has been demonstrated a role for biliary epithelial cells in portal and septal fibrosis (73). Murine biliary cells express CD14, MD-2, and TLR2, 3, 4, and 5 (74). After LPS stimulation, murine biliary cells activates the NF-κB pathway and synthesized TNFα (74). After TLR2 and TLR4 activation, CDX2 and mucus core protein-2 expression increased (75). Human biliary epithelial cells express TLR1-10 (76, 77). The progression of NAFLD in humans has been related to the increase in bile ductules, and their senescence markers. Moreover, such senescent bile ductules expressed chemotactic protein, such as MCP-1, likely responsible for HSC activation (73).

## Hepatic Dendritic Cells

Hepatic dendritic cells are the antigen-presenting cells in the liver. In inflammatory conditions, they are recruited into liver sinusoids, and migrate to periportal and pericentral areas. They express TLR4/MD-2 complex, produce inflammatory cytokines (i.e., IL-12 and TNFα), and express co-stimulatory molecules (CD40, CD80, and CD86) following several stimuli, such as LPS, peptidoglycan, poly-I:C, and cytidine-phosphateguanosine (CpG)-DNA (3). De Creus et al. (78) found a lower expression on TLR4 in hepatic dendritic cells compared to the spleen counterparts, suggesting a reduced activation of hepatic adaptive immune response; on the other hand an higher expression of TLR2 and TLR4 was shown in hepatic dendritic cells, related to an increased production of TNFα and IL-6 after LPS and peptidoglycan stimulation (79).

## Sinusoidal Endothelial Cells

Sinusoidal endothelial cells have a major role in hepatic perfusion and supply, constituting the fenestrated lining of liver sinusoids. These cells constitutively express TLR4 and CD14 and, if acutely stimulated with LPS, NF-κB activation occurs. Conversely, repetitive challenge with LPS induces a reduced activation of NF-κB pathway in these cells, associated with a reduction of CD54 expression and leukocyte adhesion (80). TLR1-9 mRNA was found in sinusoidal endothelial cells and functional expression of TLR3 has been demonstrated in controlling hepatitis B virus replication by non-parenchymal liver cells (81). The role in LPS uptake is somewhat controversial, since contrasting data were obtained (53, 82).

## PRRs: TLRs and NLRs in Liver

The innate immune system is the major contributor to acute inflammation induced by microbial infection or tissue damage (83). Currently, 10 and 12 members of the TLR family have been identified and ubiquitously expressed in humans and mice, respectively. While TLRs sense PAMPs and DAMPs at the cell surface or in endosomes, NLRs monitor the cytosolic compartment. TLRs, so named for their similarity to a protein coded by Toll-gene in the fruit fly of *Drosophila*, recognize signature motifs, PAMPs, through a conserved ectodomain with leucine rich repeats, resulting in an alertness of immune system to the presence of microbial antigens (84).

Being widely expressed in hepatic cells, TLRs can detect a range of microbial structures, promoting innate and adaptive immune responses against detected pathogens (84–87). Their signaling in the liver is associated with pathological implications in a wide range of acute or chronic liver disease. The specific recognition, rather than non-specific as previously recognized, by TLRs was discovered in mid-1990s and rewarded later by the Medicine Nobel Prize laureates Bruce A. Beutler and Jules A. Hoffmann. Physiologically, TLR signaling induces protective responses, such as pathogen clearance, regenerative mechanisms, and protection from cell death. Abnormal TLR signaling is associated to hepatic damage, endotoxin shock, organ failure, impairment of regenerative responses, fibrosis, and hepatocarcinoma (88–90).

Toll-like receptors are type I transmembrane proteins containing, a part from pathogen recognition domain, transmembrane domains, and intracellular Toll-interleukin-1 receptor (TIR) domains required for signaling transduction. Binding of TLRs leads to activation of multiple pathways from an intracellular signaling cascade, transcription of inflammatory genes, synthesis of inflammatory cytokines and interferons, and cell recruitment. It also stimulates expression of co-stimulatory molecules required to induce an adaptive immune response of antigen-presenting cells (66). However, evidence on mice deficient in each TLR has demonstrated distinctive functions of these receptors in terms of PAMP recognition and immune response (87). Microbial recognition by TLRs occurs in several cellular compartments, such as plasma membrane, endosomes, lysosomes, and endolysosomes. The localization and trafficking of TLRs within the cell is important for ligand accessibility, tolerance of self-molecules, and downstream signaling transduction (84). In fact, TLRs can be grouped into two divisions based on their subcellular localization. TLR1, 2, 4, 5, and 6 are found to be on the cell surface, while TLR 3, 7, 8, and 9 are located intracellularly and are nucleic acid sensing (91). The majority of TLR family members associate with MyD88, a common adaptor molecule, through the TIR domains, triggering inflammatory pathways. Conversely, TLR3 and TLR4 bind another adaptor protein, TIR-domain-containing adapter-inducing interferon-β (TRIF), to induce type I IFN. In order to recognize LPS, TLR4 needs to recruit LPS-binding protein, CD14, and myeloid differentiation MD-2. After TLR4 binding, the intracellular domain of TLR4 recruits TIR-domain-containing adapter protein and MyD88 for MyD88-dependent signaling, and TRIF-related adaptor molecule binds TRIF for MyD88-independent signaling (83, 92). TLR role, in the context of NAFLD/NASH onset and progression, has been particularly addressed for TLR4, TLR2, and TLR9 (93, 94).

TLR4 has been particularly studied in relation to inflammation and fibrogenesis in liver (3). Here, KC, hit by bacterial or sterile insults, contribute through TLR4 activation to many liver diseases including diet-induced liver insults. Binding of LPS to TLR4 on KC, activating NF-kB, MAPK, ERK1, p38, JNK, and IRF3, induces the production of inflammatory cytokines and type I IFN, contributing to tissue damage, increase in leukocyte infiltration, and secretion of profibrogenic cytokines. In fact inactivation of TLR4 leads to attenuation of steatosis and NASH in several models of experimental models (95, 96). Beyond TLR4, also TLR9 signaling, induced by nuclear DNA activation, has been implicated in NASH. This receptor in fact is able to bind CpG oligonucleotides, contained by DNA from gut-derived bacteria. Noteworthy, bacterial DNA was detectable in blood from mice with diet-induced NASH (93). Moreover, TLR9−/− mice were sheltered from NASH, and this protection was related to a decrease in IL-1β production by KC (93). TLR9 detrimental effect on liver was ascribed to bone-marrow-derived cells, since mice transplanted with TLR9-deleted bone marrow were protected by liver injury (97). TLR9 protective effect was also confirmed in other types of liver injury, such as I/R damage, using an inhibitory CpG sequence (97) or in acetaminophen-induced hepatocyte death, using TLR9 antagonists (98). On the other hand, TLR9 can also mediate injury-limiting pathways. It has been shown that TLR9 is involved in dendritic cells response to DNA released from cell damage. In particular, dendritic cells respond to DAMPs through the production of the anti-inflammatory IL-10 (97).

Lipopolysaccharide and other gut-derived bacterial products, secondary to bacterial translocation, can stimulate TLR in the liver (99). In this context, activation of TLRs, placed on the different hepatic cell populations, results in acute and chronic liver diseases. Evidence clearly showed a pivotal role for TLRs, gut microflora, and bacterial translocation in NASH and fibrosis. Gut microbiota has shown to modify metabolic parameter and glucose homeostasis in TLR2 knockout mice. In fact these mice acquired a phenotype reminiscent of metabolic syndrome characterized by differences in the gut microbiota (100). Moreover, the gut microbiota modifications observed were related to an increase in LPS absorption, low-grade inflammation, glucose intolerance, IR, and obesity. Interestingly, the molecular mechanisms associated to these effects involved activation of TLR4, endoplasmic reticulum stress, and activation of JNK (100).

Autophagy, a lysosome-dependent process of self-eating, has recently considered as a key regulatory pathway of the innate immune response, through effects on TLRs (101). It has been demonstrated that Atg16L1, an autophagy gene associated with susceptibility to inflammatory bowel disease, mediating autophagosome formation regulates LPS-induced intestinal inflammation. Consistently, macrophages lacking Atg16L1 produced increased amounts of the pro-inflammatory cytokines IL-1β and IL-18 in response to LPS stimulation (102). Hence, failure in macrophage macroautophagy may sustain hepatic steatosis and liver damage. Activation of macroautophagy increases the turnover of damaged proteins, peroxisomes, and mitochondria (103), reducing accumulation of harmful molecules. However, it is not known whether the impairments in autophagy, revealed in fatty livers, involve only hepatocytes or also KC. Indeed, in steatotic liver, the suppression of autophagy in KCs increases the sensitivity to endotoxin, suggesting a protective effect of autophagy on progression of NAFLD (104). PAMPs and DAMPs can also be recognized by NLR (105, 106), whose activation leads to the formation of so called inflammasome. Twenty-two NLR genes have been identified in humans thus far. The first discovered NLRs are NOD containing proteins NOD1 and NOD2, which sense bacterial peptidoglycan. Specifically, NOD1 recognizes the meso-diaminopimelic acid found predominantly in Gram-negative bacteria, whereas NOD2 detects the muramyl dipeptide present in all bacteria. NOD1/2 activation signals through MAPKs and NF-kB and induces transcriptional up-regulation of pro-inflammatory cytokines (83). Because NOD1 can recognize peptidoglycan from the gut microbiota (107), it is possible that nutrient excess is sensed by NOD1 through alteration of the gut microbiota and enhanced translocation of peptidoglycan. Several NLR members, i.e., NLRP1, NLRP3, NLRP6, and NLRC4, assemble into large multiprotein complexes called inflammasomes to control caspase-1 activity (108). Inflammasomes are sensors of endogenous or exogenous PAMPs or DAMPs that govern cleavage of effector pro-inflammatory cytokines (109). After a priming step, such as infection or injury, there is an increase in inflammasome expression, which is then triggered by PAMPs or DAMPs (110). Therefore, the inflammasome constitutes a platform for the activation of caspase-1, leading to maturation of IL-1β and IL-18 and inactivation of IL-33, regulating cell survival and death (111).

In general, activation of TLRs and NLRs induces the production of pro-inflammatory cytokines and the recruitment of immune cells, such as macrophages and T lymphocytes, in the liver, as well as other tissues, including adipose tissue, muscle, hypothalamus, pancreatic islets, and blood vessels. The resultant chronic low-grade inflammatory state promotes IR and energy imbalance and contributes to the metabolic complications of obesity, such as fatty liver disease, T2D, and atherosclerosis. The inflammasome activation has been involved in NAFLD. In particular, saturated fatty acids induce a sensitization to LPS-induced inflammasome activation and hence liver injury. As a matter of fact, dangerous signals are released by hepatocytes, contributing to inflammasome activation in immune cells (112). A critical determinant in the progression of NFLD toward NASH is the modulation of intestinal microbiota through multiple inflammasome components. Among NLR members, the NLRP3 inflammasome has also been demonstrated to play a critical role in diet-induced obesity and IR. Multiple studies using mice deficient in different components of the NLRP3 inflammasome (NLRP3 and caspase-1) have consistently shown that loss of the NLRP3 inflammasome decreases HFD-induced hepatic steatosis and inflammation (113) and improves systemic insulin sensitivity (12, 13, 114, 115). NLRs/inflammasomes play a role in the pathogenesis of steatosis/steatohepatitis induced in several experimental models through alcohol (116), acetaminophen (98), liver I/R injury (117, 118), or LPS (119, 120).

## Metabolic Complications and Pharmacological Tools

A fine regulated interaction between immunity and metabolic system exists (14). Some tissues, such as liver and adipose tissue, have preserved their analogy of structure during the evolutionary process and show a specific organization of metabolic and cellular components responsible for a direct and rapid entry into blood vessels (121).

Liver and immune system can regulate metabolic and immune functions by common regulatory molecular pathways and pathogen-sensing systems. Among these, lipid-related pathways and TLR4–NF-κβ pathway play a major role; both are activated by metabolic, nutritional, and immunological stimuli, and can influence and regulate energy balance and IR in response to changes in nutritional environment and inflammatory status (5, 122, 123).

Many observations point out a fine balance between immune and metabolic systems, identifying a main role for liver. The dysfunction between these two systems is unsafe and triggers the development of several pathologies. Overnutrition and obesity impair metabolic homeostasis, cause stress, and arise inflammatory process (124, 125), contributing to the development of the obesity-related inflammatory diseases. Conversely undernutrition and malnutrition suppress immune system and increase susceptibility to infections (14).

The condition *sine qua non* of NAFLD onset is macrovesicular steatosis or fatty liver, characterized by cellular accumulation of fat, mainly in the form of triglycerides, and sustained and amplified by the inflammatory process. The accumulation and metabolism of lipid in hepatocytes is under control of insulin or other hormones and factors that act in autocrine and paracrine manner. In particular, insulin potently inhibits hepatic endogenous glucose production, which results compromised in patients with hepatic IR (126). With its role in promoting glucose uptake, insulin has long been considered a regulator of triglyceride catabolism by inhibiting hormone sensitive lipase. Moreover, another important action of insulin is its anti-lipolytic effect in adipose tissue, which is impaired in IR, inducing an elevated release and amount of FFAs into the bloodstream. However, IR is very common in NAFLD in obese and diabetic subjects, but it has been evidenced also in non-obese and non-diabetic patients (127). However, NAFLD presents IR either in liver, impairing glucose metabolism (128), or in adipose tissue, increasing lipolysis and circulating FFAs (127). All these metabolic alterations are more evident and profound in diabetic patients, who are commonly characterized by an increase of hepatic and visceral fat and IR (129).

To date, on the basis of NAFLD pathophysiology, three therapeutic targets have been identified. The first two are metabolic approaches, regarding IR and dyslipidemia, while the third is focused on oxidative stress buffering. It is conceivable that insulin sensitizers might reverse not only IR, but also liver damage observed in NAFLD. Among these, the antidiabetic drug metformin is known to reduce hepatic glucose production and increase glucose uptake in the muscle (130). Anyway, the beneficial effects of this drug on serum ALT and liver damage do not seem to be better than those obtained from lifestyle modifications (131). Beyond metformin, the second generation thioglitazones that act as agonists of PPARγ, improve insulin sensitivity and may be considered therapeutic tools for NASH, as they are able to increase FFAs storage in subcutaneous adipocytes rather than liver and visceral fat. Moreover, they downregulate NF-kB and increase adiponectin levels (132). Anyway, even if safer than first generation drugs, thioglitazones are contraindicated in pediatric patients or in the presence of active liver disease. Furthermore, another limit is their lifelong treatment, and the possibility that, following drug cessation, steatosis might recur (133).

Drugs used in the treatment of hyperlipoproteinemias, including statins and fibrates, have been suggested beneficial in NAFLD patients, reducing the degree of hepatic steatosis but not the liver enzymes (134).

The concept of lipotoxicity and involved lipid species has been discussed in several excellent reviews (135, 136). It has been suggested that liver can accumulate the excess of FFAs as triglycerides, which are the main lipids stored in the liver of patients with NAFLD. In these patients, FFAs accumulation is mainly due to several events, among these, the reduction of fatty acid oxidation, the influx increase, and efflux reduction of fatty acids and, hence, increased *de novo* lipogenesis (137).

In physiological status, hepatocytes protect themselves by binding, transforming, catabolizing, and exporting excess FFAs. Although large epidemiological studies suggest triglyceride-mediated pathways might negatively affect this disease (138), recent evidence indicates a protective function by triglycerides. In fact, the initial increase in triglyceride synthesis could be considered a beneficial and adaptive response when hepatocytes are exposed to elevated amount of triglyceride metabolites. Therefore, hepatic fat accumulation cannot be considered as a pathology or disease, but rather, as a physiologic response to increased caloric intake (139). However, FFAs and cholesterol, especially when accumulated in mitochondria, are considered the “proactive and aggressive” lipids leading to TNFα-mediated liver damage and reactive oxygen species (ROS) formation (140). These lipids could also be present in a non-steatotic liver and act as early “inflammatory” stimuli. Inflammation results in a stress response of hepatocytes, may lead to lipid accumulation, and therefore could precede steatosis and NASH (141); interestingly, patients with NASH may have no or severe steatosis, suggesting that inflammation could take place first.

**Figure 1:**
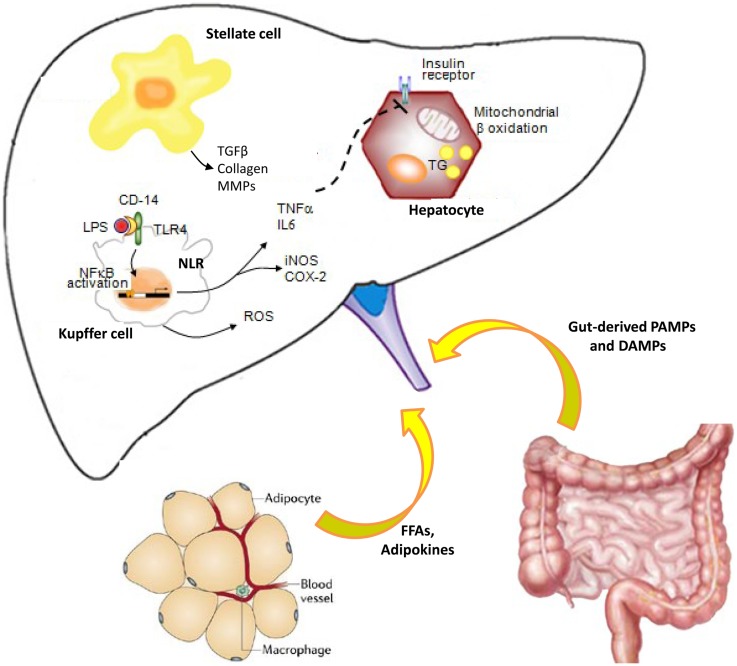
**Cross-talk between innate immune cells during liver inflammation: role of adipose tissue and gut**. Pathogen-associated molecular patterns (PAMPs) and damage-associated molecular patterns (DAMPs) bind pattern recognition receptors (PRRs), including toll-like receptors (TLRs) and NOD-like receptors (NLRs). Therefore, the liver innate immune cells initiate and sustain hepatic inflammation process, through inflammatory cytokine production. Impairment of intestinal mucosal barrier and/or dysbiosis is responsible for bacterial translocation and elevated endotoxin levels during chronic liver disease, worsening inflammation, and inducing fibrogenesis via Toll-like receptor (TLR) signaling. Kupffer cells are hit by bacterial or insults and contribute to the cytokine production and hepatic stellate cell activation to maintain the balance between inflammatory and fibrogenic signaling. Adipokines secreted from adipose tissue and inflammatory cytokines impaired hepatic insulin signaling, leading to insulin resistance. All these events lead to a vicious cycle that causes worsening of liver damage, further inflammation, and disease progression.

Lipid accumulation and subsequent lipotoxicity trigger intracellular signaling pathways, which result in pro-inflammatory cytokines that are responsible for cellular recruitment (Figure 1). As above decrypted, hepatic damage is also linked to activation of KCs, HSCs, and sinusoidal endothelial cells, which sustain inflammatory cytokine or mediator secretion. Most of these resident cells are also responsive to inflammatory factors and adipokines secreted by adipocytes. These synergic events can active vicious cycle that amplifies inflammatory process, sustaining steatosis and IR, leading to disease progression and hence worsening hepatic damage (142, 143).

The synthesis of cytokines, such as TNFα and IL-6, both involved in inflammatory and metabolic alterations, characterizes the earliest phases of different liver injury, leading to the synthesis of other cytokines that, jointly, induce cell migration and initiate healing processes, including fibrosis (144). A correlation has been found between TNF-α levels and fibrosis degree in NASH patients (145), indeed gene expression of either TNF-α or its receptor is significantly elevated in their hepatic and adipose tissues (146). Similar correlation has been found in NAFLD patients, whose circulating TNF-α are significantly elevated concomitantly with the increase in the activity score, NAS, the histologic scoring system recognized as standard reference in the evaluation and gradation of hepatic inflammation and damage (10). As well, progression of NAFLD correlates with polymorphisms in the TNF-α promoter region and serum level of soluble TNF receptor 2 (147).

Infliximab, a potent TNF-α neutralizing monoclonal antibody used in the treatment of many chronic inflammatory diseases, improves steatosis and insulin signaling both in genetic and nutritional experimental model of IR, reducing inflammation and increasing hormonal sensitivity (148, 149). However, using neutralizing anti-TNF antibodies in humans did not show a clear improvement in insulin sensitivity either in rheumatoid arthritis patients (150) or in healthy obese, but insulin resistant, patients (151). Moreover, the treatment with pentoxifylline, a TNF-α inhibitor, reduces amino-transferase serum levels and IR, measured by homeostatic metabolic assessment-insulin resistance (HOMA-IR) in NASH patients (152, 153). There is no doubt that low-grade chronic inflammation is part of NAFLD and IR but probably other pro-inflammatory cytokines are of more relevance in metabolic impairment. Thus, TNF-α blockade appears to have no efficacy on IR in humans.

Most cytokine research on obesity-related diseases has centered on IL-6, which was among the first cytokine to be implicated as a predictor or pathogenetic marker of IR and cardiovascular disease. This cytokine plays a key role in the onset of hepatic IR, which was found reduced in obese mice on high fat diet treated with anti-IL-6 antibodies (154). It has been also shown that adipose tissue-derived IL-6 regulates hepatic IR via up-regulation of SOCS3 (155). Indeed, overexpression of SOCS-3 in the mouse in the mouse liver causes IR and an increase in sterol regulatory element-binding protein (SREBP-1c) that regulates fatty acid synthesis. Conversely, inhibition of SOCS3 in obese diabetic mice improves insulin sensitivity, normalizing the increased SREBP-1c expression (124). Recently, increased expression and activity of SREBP pathway, responsible for *de novo* lipogenesis, has been associated to high fructose exposure in mice. In particular, fructose overconsumption, such as high fat diet, contributes to the development of obesity, dyslipidemia, and impaired glucose tolerance, producing advanced glycation end products (AGEs) responsible for dysfunctional proteins (156). Recently, it has shown that high AGE levels, common in western diet, exacerbate liver injury, inflammation and fibrosis via oxidative stress, cytokine synthesis (TNFα and IL-6), and HSC activation (157). So, AGE and its receptor pathway could be considered a new target for nutritional or pharmacological strategy to slow NAFLD progression.

IL-6, as well as leptin, activates AMP-activated protein kinase (AMPK) in skeletal muscle and adipose tissue. Leptin is a lipolytic hormone and pro-inflammatory cytokine with important effects in regulating body weight, metabolism, and reproductive function (158–161). Consistent with AMPK activation, IL-6 increases fat oxidation *in vitro, ex vivo*, and in humans (162, 163). A definite answer to the role of IL-6 in IR will be only possible when more clinical data will be available on the use of IL-6-neutralizing antibody in diabetic and/or IR patients. To date only two small clinical trials, however, suggest a beneficial effect. One study assessed the influence of the antibody tocilizumab in rheumatoid arthritis (164). In this study, 10/39 patients were diabetic. This biological therapy resulted in a significant reduction of hemoglobin A1c, suggesting an improvement of hyperglycemia. Similar data have been previously obtained (165). In this small study including on non-diabetic patients, tocilizumab treatment decreased HOMA index significantly and improved insulin sensitivity.

An abnormal leptin secretion may contribute to switch from insulin sensitivity to IR. Hepatic IR and high leptin concentrations are two factors that favor the entry of FFAs into mitochondria and hence the activation of PPARα. Some studies have provided information on the liver disease progression among patients with NASH, for example, those conducted in patients with lipodystrophy, a pathology characterized by reduction of peripheral fat deposition, severe hepatic steatosis, and diabetes, all conditions reversed by leptin administration (166).

Peroxisome proliferator-activated receptor-α is involved in hepatic lipid metabolism, regulating the transcription of genes encoding for enzymes involved in mitochondrial and peroxisomal β-oxidation. Indeed, PPARα and peroxisomal β-oxidation deficiency in mice confirmed the relevance of the alterations in PPARα-inducible β-oxidation in energy metabolism and in the development of NASH (167).

In NASH, abnormalities in ultrastructure of mitochondria and in peroxidation of plasma/mitochondrial membranes have been described. These modifications induce decreased mitochondrial respiration and impaired ATP generation capacity, leading to mega-mitochondria formation and cell death (168, 169). These alterations were correlated with serum TNFα, IR, and body weight. Moreover, increased mitochondrial ROS can increase hepatocyte Fas-ligand expression with a consequent activation of apoptotic mechanism of hepatocyte death (170). Lower levels of serum antioxidants are present in patients with NASH. Depletion of antioxidants via lipid peroxidation and free oxygen radical species renders the liver more susceptible to oxidative damage. In fact, treatment with various antioxidants, i.e., glutathione prodrugs (*S*-adenosylmethionine, betaine, choline), vitamin E, silymarin, decreasing production of ROS reduces steatosis in rats on choline- and methionine-deficient diets or high fat diet (171, 172). Also alpha lipoic acid, a naturally occurring thiol antioxidant, showed a hepatoprotective effect, associated with reduced expression of cytochrome P2E1, endoplasmic reticulum stress, and reduction of mitogen-activated protein kinases and NF-κB activity in mice on choline- and methionine-deficient diet (173). However, several clinical trials of putative antioxidants have been performed in patients with NASH (174–176).

Probiotics, short chain fatty acids, and intervention on gut flora positively prevent liver fat accumulation and inflammation in leptin-deficient mice (149) or rats on high fat diet (161, 177). The therapeutic effect of probiotics might be related to a variety of direct and indirect mechanisms, including modification of local microbiota, epithelial barrier function, intestinal inflammation, oxidative stress, or the modulation of immune system (178). Very recently, we studied the effect of butyrate and its synthetic amide derivative on diet-induced NAFLD in rats. The mechanisms of the therapeutic butyrate effect were related to prevention of liver inflammation and damage, steatosis, onset of IR, and imbalance of TLRs pattern in the early stage of NAFLD (177). Butyrate is known to have several distinct mechanisms of action. Among these, the epigenetic mechanism involves the hyperacetylation of histones, by inhibiting class I and class II histone deacetylases and regulating gene transcription and transcription factor activity (179).

In liver, the uncoupling protein (UCP)2 plays a key role in the adaptive response that preserves the hepatocytes viability in steatotic livers, but it also makes these cells more vulnerable to further insults. Although UCP is barely detectable in hepatocytes from normal adults (180), hepatic synthesis of UCP-2 increases after the induction of PPARα in these cells (181). The expression and activity of UCP-2 increased in hepatocytes from leptin-deficient mice (182) and from some patients with NASH or alcoholic hepatitis (183). Increased UCP-2 in mitochondria depolarizes the inner mitochondrial membrane, and augments the activity of the electron-transport chain, reducing the superoxide anion formation and calcium accumulation (184). It has been suggested that increased synthesis of UCP-2 in steatotic liver may contribute to inhibition of hepatocyte apoptosis (185), explaining why the activation of PPARα increases the survival of these cells. It is notable that cells with increased UCP activity show partially depolarized mitochondria and major vulnerability to loss of the mitochondrial inner membrane potential, when exposed to further insults (i.e., TNFα and endotoxin), with consequent depletion of ATP and necrosis (186–188).

The anatomical position of liver between the gut and the systemic circulation and hepatic cellular architecture is clearly responsible for its failure to detoxify endotoxins absorbed from portal circulation after hepatic injury. It might lead to further liver damage and escape of detrimental substances into the general circulation. In advanced stages of NAFLD, as shown in cirrhotic patients, gut-derived products can be involved in the activation of cytokine cascades and subsequent IR (189).

Not only bacterial products from the intestine but also increased translocation of bacteria may impair liver homeostasis and enhance liver inflammation through activation of the innate immune system. Bacterial translocation is defined as the migration of viable bacteria or bacterial products from the intestinal lumen to mesenteric lymph nodes or other extraintestinal organs and sites (190). Recently, it has been demonstrated that during HFD-induced diabetes in mice, commensal intestinal bacteria translocate in pathological manner from intestine toward the tissues where they trigger a local inflammation. This metabolic bacteremia was reversed by a *Bifidobacterium animalis* strain, which reduced the mucosal adherence and bacterial translocation of Gram-negative bacteria from the Enterobacteriaceae group (191).

In particular, translocated bacterial products augment the activation of hepatic immune cells through pattern recognition receptors including TLRs. Recently we demonstrated that, in a rat model of steatosis and IR induced by high fat diet, gut barrier integrity was altered (161). High fat diet induced an up-regulation of transcription and expression of TLR4 and co-receptor CD14 or MyD88 at intestinal and liver level and an imbalance of Gram-negative bacteria (Enterobacteriales and in particular *E. coli*). Gene expression analysis revealed that in the liver of rats receiving a *Lactobacillus paracasei B21060*-based synbiotic, TLR 2, 4 and 9, mRNAs expression was restored at physiological level. Therefore, the hepatic inflammatory markers, such as TNFα, IL-6, and NF-kB activation, were normalized, along with a restoration of metabolic alterations through normalization of PPAR expression and their target genes. Moreover, the synbiotic improved many aspects of IR, such as fasting response, hormonal homeostasis, glycemic control, and hepatic insulin signaling (161). Consistently with our data, Ehses et al. (192) have reported that TLR2-deficient mice are protected from IR and β cell dysfunction induced by HFD, linking TLR2 to the increased dietary lipid and the alteration of glucose homeostasis. Moreover, previous studies showed that the administration of other probiotics (i.e., *Lactobacillus* and *Bifidobacterium*) or prebiotics (i.e., inulin and oligofructose) can modulate the microbiota and improve gut permeability, thus controlling the occurrence of endotoxemia (193–197). Additional carefully designed clinical studies based on experimental mechanistic data, are need to provide clinical evidence for the efficacy in NAFLD therapy of probiotics and postbiotics (i.e., short chain fatty acids) alone or in appropriate synergistic combination with standard therapies.

## Conclusion

The anatomical position of liver between gut and systemic circulation, the hepatic cellular architecture, and the deep similarity and interconnections between innate immune cells and those resident in liver parenchyma have been highlighted, examining their interplay in the development of liver damage and chronic disease. Both cell types can modulate and interfere with critical processes implicated in metabolic disease and inflammatory pathways, initiating and sustaining liver damage and its progression. Hence, their cross-talk results to play a pivotal role in the development of NAFLD, leading to the various spectrum of NAFLD pathologies.

Further studies are needed to confirm the real beneficial effects of the therapeutic strategies to date available and their possible associations. Moreover, new insights into the different signaling pathways, evoked by innate response and liver inflammation, could provide new insights on NAFLD pathoetiology, identifying new potential therapeutic targets able, above all, to prevent its progression.

## Conflict of Interest Statement

The authors declare that the research was conducted in the absence of any commercial or financial relationships that could be construed as a potential conflict of interest.

## Abbreviations

AMPK, AMP-activated protein kinase; DAMPs, damage-associated molecular pattern; FFAs, free fatty acids; HOMA-IR, homeostatic metabolic assessment-insulin resistance; HSCs, hepatic stellate cells; IR, insulin resistance; KC, Kupffer cells; LPS, lipopolysaccharide; NAFLD, non-alcoholic fatty liver disease; NASH, non-alcoholic steatohepatitis; NLRs, NOD-like receptors; NOD, nucleotide oligomerization domain; PAMPs, pathogen-associated molecular patterns; PPAR, peroxisome proliferator-activated receptor; PRRs, pathogen recognition receptors; ROS, reactive oxygen species; TGF, transforming growth factor; TIR, Toll-interleukin-1 receptor; TLRs, toll-like receptors; TRIF, TIR-domain-containing adapter-inducing interferon-β; UCP, uncoupling protein.
